# Circulating miRNAs as Biomarkers for Neurodegenerative Disorders

**DOI:** 10.3390/molecules19056891

**Published:** 2014-05-23

**Authors:** Margherita Grasso, Paola Piscopo, Annamaria Confaloni, Michela A. Denti

**Affiliations:** 1 Laboratory of RNA Biology and Biotechnology, Centre for Integrative Biology, University of Trento, via Sommarive 9, 38123 Trento, Italy; E-Mail: margherita.grasso@unitn.it; 2 Department of Cell Biology and Neurosciences, Istituto Superiore di Sanità, Viale Regina Elena 299, 00161 Rome, Italy; E-Mails: paola.piscopo@iss.it (P.P.); annamaria.confaloni@iss.it (A.C.); 3 Neuroscience Institute, National Research Council (CNR), Padova 35100, Italy

**Keywords:** miRNAs, biomarkers, Alzheimer’s disease, Parkinson’s disease, amyotrophic lateral sclerosis

## Abstract

Neurodegenerative disorders, such as Alzheimer’s disease (AD), Parkinson’s disease (PD) and frontotemporal dementias (FTD), are considered distinct entities, however, there is increasing evidence of an overlap from the clinical, pathological and genetic points of view. All neurodegenerative diseases are characterized by neuronal loss and death in specific areas of the brain, for example, hippocampus and cortex for AD, midbrain for PD, frontal and temporal lobes for FTD. Loss of neurons is a relatively late event in the progression of neurodegenerative diseases that is typically preceded by other events such as metabolic changes, synaptic dysfunction and loss, neurite retraction, and the appearance of other abnormalities, such as axonal transport defects. The brain’s ability to compensate for these dysfunctions occurs over a long period of time and results in late clinical manifestation of symptoms, when successful pharmacological intervention is no longer feasible. Currently, diagnosis of AD, PD and different forms of dementia is based primarily on analysis of the patient’s cognitive function. It is therefore important to find non-invasive diagnostic methods useful to detect neurodegenerative diseases during early, preferably asymptomatic stages, when a pharmacological intervention is still possible. Altered expression of microRNAs (miRNAs) in many disease states, including neurodegeneration, and increasing relevance of miRNAs in biofluids in different pathologies has prompted the study of their possible application as neurodegenerative diseases biomarkers in order to identify new therapeutic targets. Here, we review what is known about the role of miRNAs in the pathogenesis of neurodegeneration and the possibilities and challenges of using these small RNA molecules as a signature for neurodegenerative conditions.

## 1. Introduction

microRNAs (miRNAs) are a family of short, single-stranded 21-22 nucleotides-long non-coding RNAs, constituting about 1% of all human genes and the most abundant class of small RNAs in animals. A repository of miRNAs from many organisms is available at the miRBase Sequence Database [1], a database of published miRNA sequences and annotation [2]. Today, more than 25,000 miRNAs have been described in organisms as evolutionarily diverse as man, worm, *Drosophila,* and also in the small plant *Arabidopsis thaliana* [3]. The discovery of miRNAs abundance in different species raised one question: what are these small non coding RNAs doing? To answer it’s important to observe their regulatory targets. The first miRNA, *lin-4*, was found in 1981 in *C. elegans* and then molecularly characterized in 1993 [4,5]. The exceptional discovery was that *lin-4* produced a pair of short RNA transcripts regulating the larval development timing by translational repression of *lin-14* [6], by sequence complementarity between *lin-4* and the 3' untranslated region (3'UTR) of *lin-14* mRNA [6,7].

### 1.1. miRNA Biogenesis and Functions

miRNAs constitute a class of gene expression modulators acting at the post-transcriptional level and fine-tuning the expression of protein-encoding genes. miRNAs modulate gene expression by cleavage or by translational repression in a sequence-specific manner [8]. Animal miRNAs have been reported to functionally target endogenous mRNAs through sites in the 3'UTR [9], but target mRNAs are repressed as efficiently by miRNA-binding sites in the 5'UTR as in the 3'UTR [10]. In 2009, a class of miRNA targets containing simultaneous 5'-UTR and 3'-UTR interaction sites has been identified [11]. Moreover, conserved miRNA target sites were also found in CDS (coding sequence) [12] and analysis of CDS-located miRNA target sites suggests that they can effectively inhibit translation [13].

miRNAs derive from long-primary transcripts (pri-miRNAs) with distinctive hairpin structures, and their processing is mediated by two endonucleases, Drosha (in the nucleus) and Dicer (in the cytoplasm). Drosha cleaves at the base of the stem to generate a ~60–100 nt hairpin pre-miRNAs [14,15]. After nuclear processing, pre-miRNA is exported into the cytoplasm by Exportin-5 (Exp5) in complex with Ran-GTP and once in the cytoplasm, it is processed by Dicer, that creates a mature miRNAs duplex of approximately 22 bp length [16,17]. It is then separated into the functional guide strand, which is complementary to the target, and the passenger strand, which is subsequently degraded. A recent study provides evidence that pre-miRNAs can give rise to three distinct endogenous miRNAs: the guide strand, the passenger strand and the loop-miR, which is an active miRNA of moderate abundance derived from the single-stranded loop region of selected pre-miRNA hairpins [18]. Complementary base-pairing of miRNA guides RISC to target mRNAs, directing degradation and translational repression via several mechanisms. miRNAs are involved in the fine regulation of several cellular processes such as development, differentiation, cell proliferation and apoptosis, and their dysregulation causes many human diseases, including cancers and neurodegenerative diseases.

### 1.2. miRNAs in the Nervous System

miRNAs are found in high abundance within the nervous system where they often display a brain-specific expression pattern and are usually found to be co-expressed with their targets. They act as key regulators of different biological functions including synaptic plasticity and neurogenesis, in which they channelize the cellular physiology toward neuronal differentiation. They can also indirectly influence neurogenesis by regulating the proliferation and self-renewal of neural stem cells.

miRNAs are dysregulated in several neurodegenerative diseases, a *spectrum* of aetiologies culminating in a final common pathway of neuronal cell death. The pathogenic mechanisms underlying neurodegeneration are complex, but the universal risk factor is aging and common themes across the disorders have been uncovered, including protein aggregation, neuroinflammation and mitochondrial dysfunction [19]. The dysfunction of miRNAs in neurodegenerative disorders and their emerging role in Alzheimer’s disease, Parkinson’s disease, amyotrophic lateral sclerosis (ALS), and Huntington’s disease (HD) pathogenesis is increasing recognized. The study of miRNAs is therefore a novel approach to understanding neurodegenerative diseases.

miRNA expression profiling of human neurological disorders has led to the identification of signatures correlated with the diagnosis, staging, progression, prognosis and response to the treatment (reviewed in [20]). However, a causal link between a specific miRNA and a disease has been established in just a few cases, and most of the mechanistic data originates from invertebrate model systems.

## 2. Neurodegenerative Diseases

Neurodegenerative diseases include several central nervous system disorders characterized by the progressive loss of neural tissues. These disorders do not have cures because the neurons of the central nervous system cannot regenerate on their own after cell death or damage. Moreover, once a neurodegenerative disease has manifested, significant neuronal loss and CNS damage will already be present, therefore early diagnosis is essential to maximize the effectiveness of disease-modifying therapies. Tremendous efforts have been made in recent years to identify the neuropathological, biochemical, and genetic biomarkers of the diseases so that the diagnosis could be established in the earlier stages.

### 2.1. Biomarkers in Neurodegenerative Diseases

Biomarkers represent essential tools for the development of therapeutic strategies to be applied in the early stages of disease. In this context, the biomarkers should be considered potent instruments for biomedical investigation because they are constituted by biochemical or molecular (genetic, epigenetic) elements expressed from a cell, tissue or fluid in a certain stage of the life or normal and/or pathological condition that can be identified, monitored and, if possible, associated to the several stages of the disease. Moreover, the identification of disease specific biomarkers at early stages could give a chance for patients to get an early treatment, which may delay the disease progression. Furthermore, the biomarkers should not only be used to help in predicting the onset of the diseases, but also to help in overseeing the rate of progression, or in responding to treatment.

For these reasons, there is an increasing effort to develop molecular diagnostic markers that meet requirements like easy accessibility, sufficiently high specificity and sensitivity, low costs and applicability by laboratories with standard equipment. The biomarkers for AD, PD and other neurodegenerative diseases such as ALS and HD, must be reliable and specific, and they should be useful in guiding us to make more accurate diagnosis and better treatment of the diseases.

Several blood, plasma, serum or cerebrospinal fluid (CSF) biomarkers born for neurodegenerative diseases have been proposed to meet these criteria. For example, for Alzheimer’s disease (AD), the most well-established measurements for detection and tracking of the preclinical and clinical stages of AD include CSF measures of Aβ42, total tau (t-tau) and phospho-tau (p-tau) [21], while for Parkinson’s disease (PD) probably the most promising is the assay of alpha-synuclein for the diagnosis and evolution of the disease [22].

Besides proteins, microRNAs (miRNAs) have also demonstrated their potential as non-invasive biomarkers from blood and serum for a wide variety of human pathologies [23]. In the last years a role for circulating miRNAs as potential biomarkers for neurodegenerative diseases has been shown. A miRNA profile specific for many neurodegenerative disorders, such as AD, Mild Cognitive Impairment (MCI) syndrome, PD, ALS and HD was found.

### 2.2. Circulating microRNAs as Biomarkers for Neurodegenerative Diseases

miRNAs’ high stability in several tissues raised the possibility of a their preservation and quantification also in biofluids. Lawrie and collegues were the first to highlight the presence of miRNAs in serum to discriminate cancer patients with diffuse large B-cell lymphoma (DLBCL) from healthy individuals [24]. Further studies demonstrated miRNAs preservation also in other biofluids such as urine, saliva, CSF and amniotic fluid [25]. Circulating miRNAs can be released by two different pathways: the first one occurs as the result of cell lysis or apoptosis in pathological conditions (tissue damage, metastasis or inflammation) by passive leakage [26]. The second process, an active transport from cells, is mediated by microvesicles (MVs) [27,28], apoptotic bodies (ABs) [29] or, in some cases, is based on microvesicle-free miRNAs, associated with various multiprotein (such as Ago2 and NPM1) [30] or lipoprotein complexes (HDL) [31].

The use of miRNAs as biomarkers has some advantages: first of all the ease of detection and extreme specificity. Unlike large RNA molecules as mRNAs, miRNAs are well preserved in formalin fixed, paraffin embedded tissues (FFPE) and in fresh snap-frozen specimens [32]. Different methods of total RNA purification (column filtration protocols, “Tri-reagents” composed by acid phenol in combination with guanidinium-thiocyanate and chloroform) and different miRNA profiling techniques (sequencing-, microarray- and quantitative reverse-transcription polymerase chain reaction (qRT-PCR)-based methods) are used for the isolation and profiling of miRNAs. Moreover, it’s important to choose a suitable normalization method (such as rRNA, U6 snRNA or a combination of different miRNAs) to remove variations and increase the accuracy of miRNAs quantification, because a reliable data analysis is essential for the study outcome.

The easiness of determining profiles of circulating miRNAs could be important for several applications, both in the research laboratory and in the clinic. Two approaches are frequently used for the selection of promising circulating miRNA as disease biomarkers. The first is based on an unbiased global approach of miRNA profiling with subsequent validation of potential biomarkers by qRT-PCR. This approach allows to profile global miRNA expression levels using relatively small amount of purified RNA in a specific and sensitive manner and gives a good opportunity to study the underlying mechanisms involved, but not previously associated, with the disease. The limitations in sensitivity and variability of this approach depend on platforms used to profile miRNAs. Moreover, this approach is less suitable for the analysis of cell-free circulating miRNA in plasma or serum because the concentrations of many circulating miRNAs are low. The second approach is based on the analysis of miRNAs that have been shown a direct connection with the disease. This approach has also certain limitations due to potential involvement of the same miRNA in diseases regarding various organs and because high expression of miRNAs in an affected organ is not necessarily accompanied by an increase of the same miRNAs in plasma or CSF [33,34].

#### 2.2.1. Alzheimer’s Disease and Circulating miRNAs

AD is the most common form of primary degenerative dementia. Clinico-pathological studies support the notion of a long “preclinical” stage of the disease; in fact it is thought that brain pathology, consisting of amyloid plaques and neurofibrillary tangles, begins 10–20 years before significant neuronal cell death and the subsequent appearance of any cognitive and behavioral symptoms [35]. Thus, fluid and imaging biomarkers could identify subjects in early symptomatic and even preclinical stages, possibly when potential treatments can best preserve cognitive function [36]. Over the last few years, a growing number of publications reported dysregulation of miRNAs expression linked to Alzheimer Disease. While altered miRNA patterns have been exhaustively investigated in AD patients’ tissue samples or cell cultures [37,38,39,40], less information is available on circulating miRNAs in AD. The first study on miRNAs as possible biomarkers for AD was published on 2007 by Schipper and colleagues who described for the first time an increased microRNA expression in Alzheimer peripheral blood mononuclear cells (PBMCs) obtained by a Microarray analysis [41]. A more recent work in blood cells described a miRNA analysis by next-generation sequencing finding a 12-miRNA signature that distinguished between AD and controls with an accuracy of 93%, a specificity of 95% and a sensitivity of 92% [42]. miRNA profiling studies were also carried out on plasma and CSF; Kumar and colleagues reported, by using a Nanostring Technology, the discovery and validation of a unique circulating 7-miRNA signature (hsa-let-7d-5p, hsa-let-7g-5p, hsa-miR-15b-5p, hsa-miR-142-3p, hsa-miR-191-5p, hsa-miR-301a-3p and hsa-miR-545-3p) in plasma, which could distinguish AD patients from normal controls with 95% accuracy (AUC of 0.953) [43], while in CSF a miRNA profiling obtained by qRT-PCR was performed in a study that described a reduced expression of hsa-miR-27a-3p in patients with Alzheimer’s disease [44]. Also Cogswell *et al.* performed miRNA analysis on CSF by qRT-PCR, identifying a set of miRNAs, so-called AD-specific miRNAs, with an expression profile in CSF similar to that of AD patients brain [45].

In addition to profiling analysis, numerous studies are based on selecting of specific miRNAs, as possible candidate biomarkers. For example Lukiw and colleagues chose to analyze miRNA associated to inflammatory signaling finding that miR-146a and miR-155 are abundant in Alzheimer’s Disease CSF and in short *post-mortem* interval brain tissue-derived extracellular fluid [46,47]. Another study on CSF showed elevated levels in Alzheimer’s disease patients for let-7b, a miRNA that activating Toll-like receptor 7, causes neurodegeneration [48]. Other works include miRNA analysis on plasma, finding levels of circulating miR-15a associate with amyloid plaque score, important for pathological diagnosis of AD [49], and miR-29a/b, miR-181c, miR-9 down-regulated in serum [50]. Moreover, it has been observed that serine palmitoyletransferase (SPT) directly regulates amyloid beta (Aβ) levels through the post-transcriptional regulation of miR-137, miR-181c, miR-9 and miR-29a/b, suggesting that SPT and the respective miRNAs are potential therapeutic targets for AD [39].

A recent study is focused on miRNA-34 family, which participates functionally in at least two signaling pathways: Bcl2 for cell survival/apoptosis and SIRT1 deacetylase for p53 or neuroprotection signalling. The study reported an increase in the circulating levels of miR-34c in both PBMC and plasma in AD compared to controls [51].

An intermediate state between normal aging and AD (and other dementias), is called Mild cognitive Impairment (MCI), usually defined as the first stage when clinical symptoms become evident [52]. Plasma miRNA biomarkers were also reported for MCI [53]: an initial pool of miRNAs was selected among known brain- and neuron- enriched miRNAs. The authors identified biomarker pairs that fall into two sets: the “miR‐132 family”, consisting in miR‐128/miR‐491‐5p, miR‐132/miR‐491‐5p and miR‐874/miR‐491‐5p and the “miR‐134 family” consisting in miR‐134/miR‐370, miR‐323‐3p/miR‐370 and miR‐382/miR‐370 with sensitivity and specificity at 79%–100% and 79%–95% respectively. In a separate longitudinal study, the identified miRNA biomarker pairs successfully detected MCI in majority of patients at asymptomatic stage 1–5 years prior to clinical diagnosis [53].

#### 2.2.2. Parkinson’s Disease and Circulating miRNAs

PD is the second most common neurodegenerative disorder and leads to progressive deterioration of motor function due to loss of dopamine-producing brain cells. It is characterized by primary symptoms such as tremor, stiffness, slowness, impaired balance and secondary symptoms including anxiety, depression, and dementia. PD is a complex disease caused by the interaction of genetic inherited and environmental acquired risk factors [54]. Mutations were found in α-synuclein (SNCA) and leucine-rich repeat kinase2 (LRRK2) genes for late-onset disease and Parkin (PARK2), PTEN Induced Putative Kinase1 (PINK1), oncogene DJ1 (DJ1) for early onset [55]. The neuropathology of PD is characterized by cellular inclusions known as Lewy bodies in neurons, the main components of which are α-synuclein, neurofilament and ubiquitin. The use of different protein biomarkers on PD patients present problems deriving from invasiveness and conflicting results among CSF proteins due to assay differences and/or blood contamination [56]. Hence, the development of circulating biomarkers for PD has great potential but surprisingly is still in its infancy. Two studies in blood cells and in total blood were recently published: the first one, using a microarray-based approach, determined the expression profile in peripheral blood mononuclear cells (PBMCs) of 19 patients and 13 controls and identified 18 significantly under-expressed miRNAs [57]. The other one, using qRT-PCR, suggested that miR-1, miR-22-5p and miR-29 expression levels in total peripheral blood allow to distinguish non-treated PD from healthy subjects, and that miR-16-2-3p, miR-26a-2-3p and miR30a differentiate treated from untreated patients [58]. miRNA profiles in plasma from PD patients have also been reported: a first study was performed by qRT-PCR in 31 patients at onset of symptoms and 25 controls and found only one significantly up-regulated miRNA, miR-331-5p [59]; a second study identified miR-1826, miR-450b-3p, miR-626, and miR-505 in 32 PD patients and 32 controls [60]. Soreq *et al.* [61] identified by NGS in blood leukocytes, 16 miRNAs significantly altered in PD patients compared to healthy controls, including miR-16, miR-20a and miR-320. Eleven miRNAs were modified following deep-brain stimulation (DBS) treatment, five of which were changed inversely to the disease-induced changes [61].

#### 2.2.3. Amyotrophic Lateral Sclerosis and Circulating miRNAs

Amyotrophic lateral sclerosis (ALS) is a rare, devastating and fatal neurological disorder that causes weakness, atrophy, paralysis and eventually respiratory failure due to the selective degeneration of the neurons responsible for voluntary movements. ALS is a complex pathology, whose causal factors are currently unknown. Emerging evidence suggests a combined involvement of genes and environmental factors.

During the past decade there has been a large increase in the number of ALS biomarker studies using CSF as well as blood [62]. Most of these studies have examined changes of individual proteins in the CSF of ALS versus healthy controls or other neurologic disease subjects, typically using a gel-based system or ELISA [63]. However, most are limited by the number of samples used in the analysis, choice of control subjects, and typically the lack of verification in a separate cohort of patients. At present, CSF candidate biomarkers in ALS can be grouped into those that reflect neuronal loss and those indicative of neuroinflammatory (glial) processes. Regarding miRNAs as possible biomarkers in ALS, we have, so far, little knowledge. One study was in fact performed on miRNA profiling of ALS subjects, by analyzing 911 human miRNAs using microarray technology in leukocytes [64]. The study reported a profile of identified eight miRNAs that were significantly up- or downregulated in sALS patients as compared to healthy controls. In parallel, an analysis on sorted CD14+CD16– monocytes from ALS patients was performed. It is known that inflammatory monocytes were activated and that their progressive recruitment to the spinal cord correlates with neuronal loss. This study had showed a profile constituting an inflammatory signature of 56 miRNAs significantly affected in CD14+CD16– monocytes that could serve as a biomarker for disease stage or progression [65]. Despite the two analyses were performed with the same technical approach, the populations of dysregulated miRNAs found in leucocytes [64] and monocytes [65] are not overlapped and comparable.

Moreover, little or no studies were performed about circulating miRNA in plasma or serum of patients with various other neurodegenerative diseases, such as HD [66], or Frontotemporal dementia. However, it is interesting to note that miR-34b, whose downregulation is coupled to a decrease in the expression of DJ1 and Parkin, two proteins associated to familial forms of PD [67], is also up-regulated in response to Huntingtin (mHTT) mutant in both pluripotent and neuronally differentiated human cells and in human plasma, suggesting a miR-34b role as biomarker for HD [66]. Nevertheless, the study of circulating miRNA in plasma/serum for diagnosis of neurodegenerative disorders needs again more detailed investigations.

Overall, there is a lack of overlap (refer to Table 1, Table 2 and Table 3) and little concordance among these miRNA profiles both for the specific disease and when comparing different neurodegenerative disorders, which highlights the difficulties of analyzing different sample types and comparing different methodologies.

**Table 1:** Alzheimer’s Disease and circulating miRNAs.

miRNA	Sample	Experimental approach	Pilot study	Validation study	Reference
Increased expression of miR-34a and miR-181b	PBMC	miRNA profiling	Microarray AD: 16 NEC: 16	Taqman^®^ miRNA qRT-PCR	[41]
12-miRNA signature: miR-112, miR-161, let-7d-3p, miR-5010-3p, hsa-miR-26a-5p, hsa-miR-1285-5p, and hsa-miR-151a-3p upregulated; miR-103a-3p, miR-107, miR-532-5p, miR-26b-5p, let-7f-5p downregulated	Peripheral blood	miRNA profiling	Next Generation Sequencing AD: 48 CT: 22	SYBR qRT-PCR AD: 94 MCI: 18 MS: 16 PD: 9 DEP: 15 BD: 15 Schiz: 14 CT: 21	[42]
7-miRNA signature: hsa-let-7d-5p, hsa-let-7g-5p, hsa-miR-15b-5p, hsa-miR-142-3p, hsamiR-191-5p, hsa-miR-301a-3p and hsa-miR-545-3p	Plasma	miRNA profiling	Nanostring AD: 11 MCI: 9 CT: 20	Taqman^®^ miRNA qRT-PCR AD: 20 CT: 17	[43]
Reduced expression of miR-27a-3p	CSF	miRNA profiling	qRT-PCR AD CT	qRT-PCR AD: 35 CT: 37	[44]
60 miRNAs differentially regulated between the different Braak stages, including Let-7 family members	CSF	miRNA profiling	Taqman^®^ miRNA qRT-PCR Braak stage V AD: 10 Braak stage I non demented controls: 10		[45]
Elevated levels of miR-146a and miR-155	CSF and ECF	Candidate miRNAs	Microarray AD: 5 CT: 5		[47]
Significant increase in miR-9, miR-125b, miR-146a, miR-155	CSF and ECF	Candidate miRNAs	Microarray AD: 6 CT: 6		[46]
Elevated levels of let-7b	CSF	Candidate miRNAs	Taqman^®^ miRNA qRT-PCR AD: 13 CT: 11		[48]
miR-15a associated with amyloid plaque score	Plasma	Candidate miRNAs	Microarray	qRT-PCR	[49]
miR-29a/b, miR-181c, and miR-9 down-regulated	Serum	Candidate miRNAs	SYBR qRT-PCR AD: 7 MCI/Early AD: 7 CT: 7		[50]
Increased levels of miR-34c	PBMC or cell-free plasma	Candidate miRNAs	Taqman^®^ miRNA qRT-PCR AD: 110 CT: 123		[51]
Circulating brain-enriched miRNAs: “miR‐132 family” and “miR‐134 family”	Plasma	Candidate miRNAs	TaqMan^®^ miRNA qRT-PCR MCI: 10 CT: 10	TaqMan^®^ qRT-PCR MCI: 20 AD: 20 CT: 20 CY: 20	[53]

(PBMC: Peripheral Blood Mononuclear Cells; NEC: Normal Elderly Controls; AD: Alzheimer Disease; CT: Control; MCI: Mild Cognitive Impairment; MS: Multiple Sclerosis; PD: Parkinson Disease; DEP: Major Depression; BD: Bipolar Disorder; Schiz: Schizophrenia; CSF: Cerebrospinal Fluid; ECF: brain tissue-derived extracellular fluid; CY: Young Control).

**Table 2:** Parkinson’s Disease and circulating miRNAs.

miRNA	Sample	Experimental approach	Pilot study	Validation study	Reference
18 significantly under-expressed miRNAs	PBMC	miRNA profiling	Microarray PD: 19 CT: 13		[57]
miR-1, miR-22-5p and miR-29 distinguish non-treated PD from healthy subjects	Total peripheral blood	miRNA profiling	qRT-PCR untreated PD: 8 treated PD: 4 early-onset PD: 7 CT: 8		[58]
miR-16-2-3p, miR-26a-2-3p, miR30a differentiate treated from untreated patients	Total peripheral blood	miRNA profiling	qRT-PCR untreated PD: 8 treated PD: 4 early-onset PD: 7 CT: 8		[58]
miR-331-5p upregulated	Plasma	miRNA profiling	Taqman^®^ miRNA qRT-PCR PD: 31 CT: 25		[59]
miR-1826, miR-450b-3p, miR-626, and miR-505	Plasma	miRNA profiling	Agilent microarray PD: 32 CT: 32	Taqman^®^ miRNA qRT-PCR treated PD: 20 untreated PD: 10 PSP: 5 MSA: 4 CT: 4	[60]
16 miRNAs (including miR-16, miR-20a and miR-320) modified in patients pre-DBS	Leukocytes	miRNA profiling	Next Generation Sequencing PD pre-DBS: 3 PD post-DBS: 3 CT: 6		[61]

(PD: Parkinson Disease; PSP: Progressive Supranuclear Palsy; MSA: Multiple System Atrophy; DBS: Deep Brain Stimulation).

**Table 3:** Amyotrophic Lateral Sclerosis and circulating miRNAs.

miRNA	Sample	Experimental approach	Pilot study	Validation study	Reference
8 miRNAs (miR-451, miR-1275, miR-328, miR-638, miR-149, miR-665 and miR-338-3) significantly up- or downregulated	Leukocytes	miRNA profiling	Microarray ALS: 8 CT: 12	TaqMan^®^ miRNA qRT-PCR ALS: 14 CT: 14	[64]
Expression of ALS-specific miRNA inflammatory signature	Monocytes	miRNA profiling	Microarray ALS: 8 MS: 8 CT: 8		[65]

ALS: Amyotrophic lateral sclerosis.

## 3. Neurodegenerative Diseases Meet Cancers in a miRNA World

Although cancer and neurodegenerative diseases are two distinct pathological disorders, one due to an increased proliferation and the other to premature cell death, emerging evidences indicate that these two types of disease share common mechanisms of regulation. Recent studies show an involvement of miRNAs in the pathology of both diseases, indicating a convergence at the post-transcriptional level. It has been demonstrated that individual miRNA can be involved in the development of both diseases by two different mechanisms: common pathways can be regulated by multiple miRNAs or distinct pathways can be controlled by the same miRNA (reviewed in [68]).

In the first group, some miRNAs were identified as regulators of genes involved as in neurodegenerative disorders as well as in cancer (refer to Table 4). let-7 family, miR-17-5p, miR-20a and miR-106b have a role in the regulation of amyloid precursor protein (APP). An association of APP levels with Alzheimer disease (AD) has been demonstrated in several studies [69,70,71,72], with evidences showing that APP is concentrated at neuronal synapses and in AD-associated amyloid plaques following proteolysis. On the other hand, APP is also over-expressed in cancer of oral cavity, esophageal, pancreatic, neuroendocrine, thyroid, and colorectal cancers [73,74,75,76].

Another family of miRNAs involved in AD is miR-29a/b-1 family. Indeed, these miRNAs showed a role in the post-transcriptional regulation of β-secretase 1 (BACE1), an aspartic acid protease potentially important to the pathophysiology of AD [77], with evidences suggesting that high expression of BACE1 can give rise to inappropriate cleavage of APP and as a consequence to an increased load of amyloid β-peptides in patients with sporadic AD [78]. In another study it has been demonstrated that BACE1 could be the secretase responsible for IR-induced cleavage of ST6GalI, and this cleavage could decrease ST6GalI-mediated cancer cell migration [79].

miR-21 and miR-106b were shown to be involved in the regulation of phosphatase and tensin homolog (PTEN), a protein implicated in the development of Parkinson’s disease (PD) through activation of two genes involved in neural protection from oxidative stress, PTEN-induced putative kinase (PINK1), and parkinson disease 7 (PARK7, also known as DJ-1) [80]. About the role of these miRNA in cancer regulation, what is known is that PTEN is a tumour suppressor, mutated in sporadic and inherited tumours [81] and involved in the inhibition of PI3K/Akt/mTOR signaling, a pathway leading to enhanced cell survival and growth in a number of human cancers [82].

**Table 4:** Pathways regulated by multiple miRNAs (common to cancer and neurodegenerative diseases).

miRNA	Target genes	Expression in cancer	Expression in neurodegenerative disorders	Reference
let-7 family, miR-17-5p, miR-20a miR-106b	**APP**	Oncogene, over-expressed in oral cavity, esophageal, pancreatic, neuroendocrine, thyroid, and colorectal cancer	Concentrated at neuronal synapses and in AD-associated amyloid plaques	[69,70,71,72,73,74,75,76]
miR-29a/b-1 family	**BACE1**	Decrease of ST6GalI-mediated cancer cell migration	High expression in patients with sporadic AD	[78,79]
miR-21 miR-106b	**PTEN**	Tumour suppressor, mutated in sporadic and inherited tumours	Implicated in PD through activation of PINK1 and PARK7	[80,81,82]
miR-34b/c	**PARK2**	Tumour suppressor, mutated in glioblastoma, lung cancer and colon cancer	Reduced in PD brain samples displaying strong miR-34b/c downregulation	[67,83]

(APP: Amyloid Precursor Protein; BACE1: β-secretase 1; PTEN: Phosphatase and Tensin Homolog; PARK2: Parkin).

Another important gene involved in PD is Parkinson protein 2 E3 ubiquitin protein ligase (PARK2). Inactivating somatic mutations and frequent intragenic deletions of PARK2 lead to human malignancies and PARK2 mutations in cancer occur in the same domains as the germline mutations causing familial PD. PARK2 mutations in cancer decrease PARK2’s E3 ligase activity, compromising its function in the ubiquitination of cyclin E and resulting in mitotic instability. On the basis of these data, PARK2 could be a gene causing neuronal defects when altered in the germline, and instead contributing to oncogenesis when mutated in non-neuronal somatic cells [83]. It has been demonstrated that DJ1 and PARK2 are indirect targets of miR-34b/c: DJ1 and PARK2 expression is reduced in PD brain samples displaying strong miR-34b/c downregulation. In brain areas with neuropathological damage at clinical stages of PD, miRNA expression profiles reveal decreased expression of miR-34b and miR-34c [67].

In the second group, there are some miRNAs involved in the pathology of both neurodegeneration and cancer by targeting distinct pathways that are specific for each disease (refer to Table 5). miR-29 family members are demonstrated to be involved in the regulation of neuron navigator 3 (NAV3) in the context of AD [84]. Studies in lung cancer and acute myelogenous leukemia have shown that the same family of miRNA directly target DNA methyltransferases DNMT3A and DNMT3B [85,86].

Another miRNA family, the let-7 family, in addition to its involvement in the regulation of APP in AD in *C. elegans* [87], seems to have a role in many types of cancer through its regulatory effects on a variety of target proteins such as Ras, HMGA2 and Myc [88,89,90,91].

The third family with an involvement in the regulation of AD [92] and PD [93] is miR-106b family promoting also cell-cycle progression and inducing G1-arrest through negative regulation of the cyclin-dependent kinase inhibitor 1A (p21/CDKN1A) [94]; moreover, in breast cancer cells this miRNA family is able to target breast cancer metastasis suppressor 1 (BRMS1) and RB1 [95].

With respect to PD, miR-124a and miR-133b by suppressing the expression of the PITX3 transcription factor [96] have shown a role in the regulation of this disease. In HeLa cells, over-expression of miR-124a induces a neuron-specific expression profile [97], and some of the identified downregulated genes were also deregulated in dopamine neurons isolated from affected areas of patients brains with idiopathic PD [98]. miR-124a has been also related to tumorigenesis through its modulation of the expression of the cyclin-dependent kinase 6 (CDK6) oncogene and the retinoblastoma (RB1) tumor suppressor gene [99]. miR-133b, down-regulated in a number of tumor types, including esophageal, lung, and colon cancers [100,101,102,103], targets myeloid cell leukemia 1 (MCL1, also known as BCL2L3) and BCL2-like 2 (BCL2L2) [101], oncogenic actinbinding factors like fascin homolog 1 (FSCN1) [102], and the met proto-oncogene receptor tyrosine kinase (MET) [103].

The ease of detection and quantification of miRNAs in blood and other biofluids paves the way to use miRNAs involved in both neurodegenerative disease and cancer as therapeutic targets or agents in the treatment of both diseases, while miRNAs differentially expressed in these two different diseases as diagnostic markers.

**Table 5:** Pathways controlled by the same miRNA (distinct in cancer and neurodegenerative diseases).

miRNA	Disease	Target genes	Reference
miR-29 family	Lung cancer and acute myeloid leukemia	DNMT3A DNMT3B	[85,86]
	AD	NAV3	[84]
let-7 family	Lung cancer (Ras); Benign mesenchymal tumors and lung cancers (HMGA2); Burkitt lymphoma (Myc)	Ras HMGA2 Myc	[88,89,90,91]
	AD	APP	[87]
miR-106b family	Several cancers (p21/CDKN1A); breast cancer (BRMS1, RB)	p21/CDKN1A BRMS1 RB	[94,95]
	AD and PD	APP PTEN	[92,93]
miR-124a	Colon cancer	CDK6 RB	[99]
	PD	Unknown	[97,98]
miR-133b	Esophageal, lung and colon cancers	MCL1 BCL2L2 FSCN1 MET	[100,101,102,103]
	PD	PITX3	[96]

(DNMT3A: DNA Methyltransferase 3A; DNMT3B: DNA Methyltransferase 3B; NAV3: Neuron Navigator 3; HMGA2: High Mobility Group AT-hook Protein 2; p21/CDKN1A: Cyclin-Dependent Kinase Inhibitor 1A; BRMS1: Breast Cancer Metastasis Suppressor 1; RB: Retinoblastoma; CDK6: Cyclin-Dependent Kinase 6; MCL1: Myeloid Cell Leukemia 1; BCL2L2: B-Cell CLL/Lymphoma 2 like 2; FSCN1: Fascin Homolog 1).

## 4. Conclusions

The data reviewed here suggest that the analysis of circulating miRNAs is a highly promising approach for developing minimally invasive screening tests for neurodegenerative disorders. The advances in the analyses of circulating miRNA summarized here could lead to more efforts toward using miRNAs as biomarkers for neurodegenerative diseases in order to ease an early diagnosis and identify new therapeutic targets.
